# Enzymatic Metabolism of Flavonoids by Gut Microbiota and Its Impact on Gastrointestinal Cancer

**DOI:** 10.3390/cancers13163934

**Published:** 2021-08-04

**Authors:** Raghad Khalid AL-Ishaq, Alena Liskova, Peter Kubatka, Dietrich Büsselberg

**Affiliations:** 1 Weill Cornell Medicine-Qatar, Education City, Qatar Foundation, Doha 24144, Qatar; rkmalishaq@hotmail.com; 2 Department of Obstetrics and Gynecology, Jessenius Faculty of Medicine, Comenius University in Bratislava, 036 01 Martin, Slovakia; esova5@uniba.sk; 3 Department of Medical Biology, Jessenius Faculty of Medicine, Comenius University in Bratislava, 036 01 Martin, Slovakia; peter.kubatka@uniba.sk

**Keywords:** gastrointestinal cancer, flavonoids, gut enzymes, microbiome, anticancer

## Abstract

**Simple Summary:**

Flavonoid’s consumption is reported to impact GI cancer progression positively. As 90% of flavonoids consumed, undergo metabolism and conversion by the human gut microbiome, understanding their enzymatic bioconversion and metabolism could advance the current knowledge of their anticancer activities. While it is reported that specific flavonoids target cancer-related pathways such as apoptosis, inflammation and cellular proliferation, efforts are required to assess the possibility of combining those specific flavonoids together or with current treatment such as chemotherapy and evaluate their effect on the pathogenesis of GI cancer. Additionally, Studies aimed to standardize flavonoids administered concentration, purification and isolation methods are required.

**Abstract:**

Gastrointestinal (GI) cancer is a prevalent global health disease with a massive burden on health care providers. Internal and external factors such as obesity, smoking, diet (red meat), low socioeconomic status and infection with *Helicobacter pylori* are the critical risk factors of GI cancers. Flavonoids are natural phenolic compounds found abundantly in fruits and vegetables. Upon ingestion, 90% of flavonoids consumed require further enzymatic metabolism by the gut microbiome to enhance their bioavailability and absorption. Several epidemiological studies reported that consumption of flavonoids and their enzymatic conversion by gut microbes is strongly associated with the reduced risk of GI cancer development. This review summarizes the current knowledge on the enzymatic conversion of flavonoids by the human gut microbiome. It also addresses the underlying anti-GI cancer effects on metabolic pathways such as apoptosis and cellular proliferation. Overall, metabolites produced from flavonoid’s enzymatic conversion illustrate anti-GI cancer effects, but the mechanisms of action need further clarification.

## 1. Introduction

### 1.1. Gastrointestinal Cancer

Cancers are one of the major causes of disabilities and death worldwide [1]. It is a heterogeneous disease triggered by the impairment of cellular function and homeostasis [2]. Among all organ cancers, gastrointestinal (GI) cancers account for many malignancies and are considered a challenging public health problem with a substantial medical and economic burden worldwide [3]. “GI cancers” is a term used to describe cancers that affect the digestive system, including colorectal cancer (CRC), gastric cancer (GC), esophageal cancer (EC), hepatocellular carcinoma (HCC) and pancreatic cancer (PC) [4]. Factors such as obesity, smoking, diet (red meat), low socioeconomic status and infection with *Helicobacter pylori* are the critical risk factors of GI cancers [5]. Depending on the type of cancer, symptoms and signs include fatigue, weight loss, abdominal pain, anorexia, dysphagia and hematemesis [6,7]. Cancer progression in the body results from the loss of apoptotic functions and the uncontrolled cell growth and differentiation leading to neoplastic cell expansion [8]. Critical pathways involved in GI cancer biology include intrinsic and extrinsic apoptotic pathways, protein kinase B (AKT) and phosphatidylinositol 3-kinase (PI3K), which plays a role in cellular proliferation, nuclear factor kappa (NF-κB), that plays a role in the progression of cancer by triggering cellular inflammation, and epithelial-mesenchymal transition (EMT) which promote cellular invasion and metastasis [9]. More efforts are needed to understand the underlying causes of these pathways on the progression of GI cancer.

### 1.2. Flavonoids and Cancer

Flavonoids are natural products ubiquitously found in fruits and vegetables [10]. They are biologically active secondary plant metabolites with multiple health benefits [11]. Structurally, flavonoids consist of 15 carbon atoms and two benzene rings (A and B) linked by an oxygenated heterocyclic C ring. Depending on the structure of the C ring, the functional group on the ring, and the attachment site to the C ring, flavonoids could be further classified into six subclasses: flavonols; flavones; flavanones; iso-flavones; flavan-3-ols; and anthocyanosides, where each class differs in the degree of substitution and hydroxylation [12].

Recent epidemiological studies reported that regular consumption of flavonoids is strongly associated with the reduced risk of GI cancer development [13]. Another study reported an inverse association between the total intake of flavonoids and gastric adenocarcinoma [14]. As for colon cancer, a meta-analysis review was performed on 17 epidemiological studies to assess the impact of soy isoflavone consumption on colorectal cancer [15]. The results showed that soy isoflavone consumption reduced the risk of colorectal cancer by 23%, with a relative risk value of 0.77. Among the 17 reviewed studies, three studies used tofu as an exposure, two used soybeans, two used bean curd, four used soy products, one used genistein and five used a combination of soy products and isoflavone. In addition, results from an in vitro study demonstrated the ability of flavonoids to inhibit the growth of colon cancer cells [16]. Additionally, an inverse association between colon cancer and flavonoid intake (anthocyanidins, flavanones and flavones) was observed in a case-control study performed on the Chinese population (3264 participants) [17]. More studies are required to explain the association observed and address the possible protective effect of flavonoids on GI cancer. 

### 1.3. Flavonoids Metabolism in the Gut 

**Figure 1:**
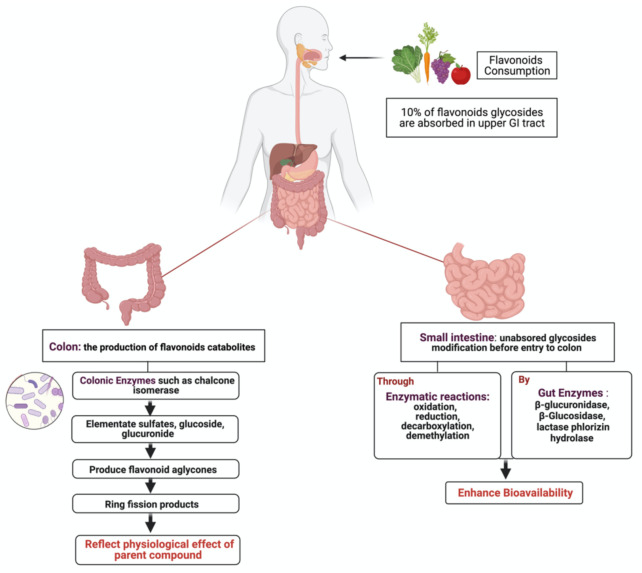
Illustration of flavonoids metabolism by gut microbiota. After the ingestion of flavonoid, a small percentage becomes absorbed in the upper GI tract, while the rest enters the colon for further modification to produce ring fission products that reflect the physiological effects of the parents’ compound. Created with BioRender.com (accessed on 3 August 2021).

Dietary flavonoids exist either as aglycones with no attached sugars or glycosides with attached sugars (most common) [18]. Depending on the type of the attached sugar moiety, extensive metabolism by gut microbiota and host tissue occurs [19]. Global interest in flavonoids products produced by intestinal microbiota and their potential physiological role has recently increased [20]. After ingestion, approximately 10% of flavonoids glycosides are absorbed in the upper GI tract, where the remaining 90% pass through the small intestine to reach the colon as non- metabolized and non-absorbed flavonoids [21] (Figure 1). Unabsorbed flavonoids undergo enzymatic modification in the small intestine, such as oxidation, reduction and decarboxylation, as a preparation step before colon entry. Once in the colon, colonic enzymes produced by microbiota eliminate glycosides to produce flavonoid aglycones, which are further metabolized into ring fission products [22]. It is proposed that these catabolites produced with lower molecular weight reflect the physiological effect of their flavonoid parent compounds [23]. In the liver, further conjugation of metabolites occurs to produce sulfate derivatives, excreted through bile and urine [24]. 

**Figure 2:**
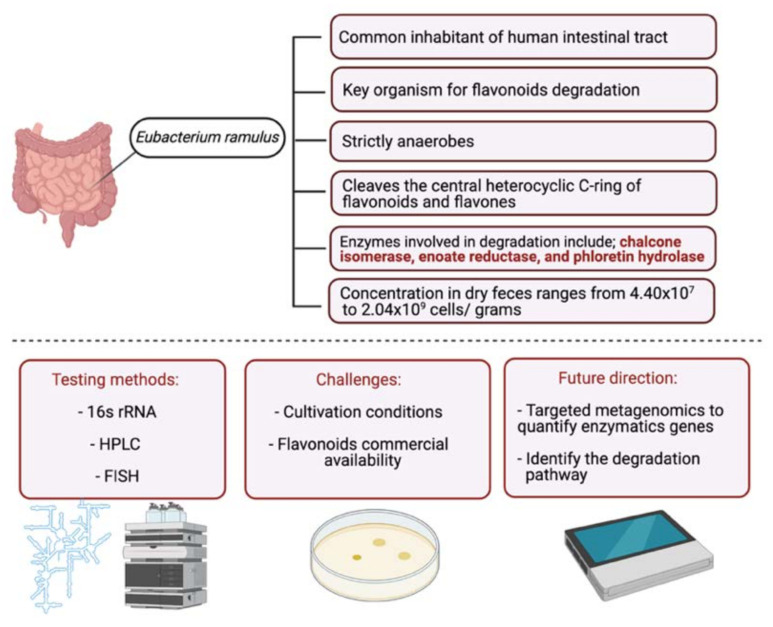
Schematic illustration of *E. ramulus*, a flavonoid degrading bacterium. The figure is divided into two parts. In the upper part, information about the bacteria is stated, and in the lower part, common testing methods, challenges with studying bacteria and efforts required to improve the field are listed. Created with BioRender.com (accessed on 3 August 2021).

In the intestinal tract, several bacteria are capable of degrading and metabolizing flavonoids. *Eubacterium ramulus*, isolated from fecal samples, degrade various flavonoids in vitro enzymatically [25]. A study on 28 healthy participants investigated the impact of selected flavonoids (quercetin and rutin) on the flavonoid degrading bacterium, *E. ramulus*. The participants were given a rich flavonoid diet, and their fecal samples were collected. *E. ramulus* was detected in the fecal samples of all participants with a concentration ranging from 2.3 × 10^8^ to 1.55 × 10^9^ cells∙g^−1^ dm. These results suggest that the administered dietary flavonoids could act as a substrate for *E. ramulus* resulting in the formation of flavonoid degradational products [26]. More studies are required to investigate the relationship between *E. ramulus* and other flavonoid groups (as the study investigated only quercetin and rutin) and classify other microbial species (Figure 2).

This review evaluates and summarizes studies that report flavonoids metabolism by gut microbiota enzymes and addresses their impact on the development of GI cancer pathways. Additionally, gaps in the current literature were identified.

## 2. Search Strategy and Selection Criteria

A comprehensive search on databases such as Medline, PubMed and Scopus was carried out for studies published from 2000 using the search terms “flavonoids”, “microbiota”, “flavonoids AND microbiota”, “flavonoids metabolism”, “flavonoids ring fission”, “flavonoids AND GI cancer”, “gut microbiota enzymes for flavonoids”, “flavonoids OR flavonoids subclasses AND GI cancer”, “flavonoids subclasses AND gastric cancer” and “flavonoids subclasses AND colorectal cancer”. The search yielded 2338 articles, and in this article, we selected 182 articles and analyzed them in detail. Eligible studies included in this review are in vivo, in vitro and clinical trials papers addressing flavonoids metabolism by human gut microbiota and the possible underlying anti-GI cancer effects. Duplicates and studies with other flavonoid metabolism mechanisms were excluded.

## 3. Metabolism of Flavonoids by Gut Microbiota

As discussed in Section 1.3, flavonoids require further modification and transformation by the gut microbiota to enhance their bioavailability and biological activities. The regulation of specific reactions catalyzed by bacterial enzymes of gut microbiota may offer new clinical opportunities to enhance the efficacy of numerous drugs, including flavonoid-derived substances [27]. Scientific databases demonstrate comprehensive results on the biological activities of flavonoids metabolites generated by phase I and phase II metabolic processes linked with gut microbiota-mediated biotransformations [28]. In this section, flavonoids will be divided into their respective groups to discuss common microorganisms, chemical reactions involved in the transformation, the impact of the metabolism of biological properties of the flavonoids. Table 1 summarizes the main findings in the literature.

### 3.1. Flavonol

Flavonols constitute a significant class of flavonoids and are divided into six groups. The unsaturated carbon ring at C2–C3 characterizes them. They are most prevalent in grapes, lettuce, kale, onions and barriers [96].

#### 3.1.1. Rutin

Rutin is a glycoconjugate form of quercetin, representing the most consumed flavonol in the United Kingdom and Europe (3.75%) [29]. Rutin exerts multiple health benefits as it has antioxidant and anti-neurodegenerative properties [30]. In the upper intestinal tract, rutin is poorly absorbed, and it accumulates in the large intestine. Several gut microbes such as *Lactobacillus acidophilus*, *Lactobacillus plantarum* and *Bifidobacterium dentium* possess alpha-rhamnosidase activities in the colon which hydrolyzes rutin, removing sugar moiety and permitting aglycone absorption [31]. A study investigated the metabolism of rutin by the human gut microbiota using anaerobic incubations of freshly collected stool samples from 10 healthy participants [32]. Products from rutin conversion (quercetin-3-glucoside and quercetin) were detected in all samples with drastic variation in the concentration, suggesting inter-individual variation in capability or preference for the metabolism of rutin. Additionally, *Enterobacteriaceae* were associated with quercetin-3-glucoside production, while *Lachnospiraceae* with quercetin production from rutin metabolism. In addition, the study reported that the alpha diversity in the active subset of the microbial community is low compared to the whole community, which suggests that part of the microbial community is metabolically activated by rutin.

Differential proteomics was used to investigate the impact of rutin on *Lactobacillus acidophilus* biological activities [33]. It regulated the level of protein expression involved in the stress response mechanism.

More efforts are required to investigate the role of gut metabolism in the bioavailability and absorption of rutin and the possible bacteria-polyphenols interaction activities.

#### 3.1.2. Fisetin

Fisetin is a bioactive flavonol with chemoprotective and anti-inflammatory properties [34]. The metabolism and bioconversion of fisetin by gut microbiota are poorly discussed in the literature. A study published in 2013 investigated the interaction of multiple flavonoids, including fisetin and probiotic bacteria [35], showed that flavonoids could promote the expression of nitric oxide produced by *Bifidobacterium adolescentis* suggesting that flavonoids may have a prebiotic-like effect on the activities of *B. adolescentis*.

#### 3.1.3. Kaempferol

A nontoxic dietary flavonol possesses antioxidant, anti-inflammatory, anti-microbial and anticancer abilities [36]. In the intestinal tract, kaempferol undergoes degradation reactions by gut microbiota to produce absorbable metabolites [37]. Variations in concentration and duration required to produce these metabolites were observed in a study that measured the interaction of phenolic compounds with gut microbiota using fecal samples from healthy participants [38]. Additionally, a study investigated the effects of *Lactobacillus paracasei* A221 on the bioavailability and functionality of kaempferol glycoside [39], showing that treatment of *Lactobacillus paracasei* A221 on the intestinal barrier model improved barrier integrity. In addition, the direct bioconversion of kaempferol by this strain seems to enhance the beneficial health properties of kaempferol metabolites, suggesting that *Lactobacillus paracasei* A221 is capable of modulating and enhancing the bioavailability and functionality of kaempferol.

#### 3.1.4. Quercetin

Quercetin is an antioxidant flavonol with critical biological activities on cellular transduction and progression pathways regulation [40]. It presents mainly as a glycoside (conjugated to sugar moieties) rather than an aglycone, which reduces its bioavailability [41]. After ingestion, quercetin reaches the small intestine to undergo deglycosylation by Lactate phlorizin hydrolase enzyme, yielding quercetin aglycone [42]. It is metabolized by multiple gut microbes such as *Bacteroides fragilis*, *Clostridium perfringens*, *Eubacterium ramulus*, *Streptococcus S-2 Lactobacillus L-2*, *Bifidobacterium B-9* and *Bacteroides JY-6* to produce 3,4-dihydroxyphenylacetic acid, 3-(3-hydroxyphenyl) propionic acid, 3,4-dihydroxybenzoic and 4-hydroxybenzoic acid metabolites [43,44]. Some of these metabolites have biological activities such as free radical scavenging activities observed with 4-hydroxybenzoic acid administration [44]. Concentration, abundance and positive relationship between gut microbes and quercetin metabolism vary depending on food intake, as reported in a study performed on elderly Japanese participants [45].

#### 3.1.5. Isorhamnetin

This is a flavonol is found abundantly in medical plants and has anti-obesity, anti-cancer and anti-diabetic activities [46]. To understand isorhamnetin’s metabolic pathway and metabolites, fecal samples from a healthy female participant and isolated different bacterial colonies were collected [47]. Using ultra-performance liquid chromatography technique coupled with the MetabolynxTM software, the metabolic profile of the samples was analyzed. In this case, 100 bacterial colonies were identified (68 *Escherichia*, 16 *Enterococcus* and 16 *Bacillus*). These bacterial colonies were incubated anaerobically with isorhamnetin-3-O-neohesperidoside to measure metabolites production. Four metabolites were observed: isorhamnetin-3-Oneohesperidoside (M1), isorhamnetin-3-O-glucoside (M2), isorhamnetin (M3) and quercetin (M4). The study suggested the metabolic pathway to be as follows; deglycosylation occurs first to produce isorhamnetin-3-O-glucoside and subsequently produce isorhamnetin, then demethylation occurs to produce quercetin. Additional studies are required to support these findings and to highlight important enzymes involved in the mechanism.

### 3.2. Flavanones

Flavanones are known as dihydroxyflavones and are recognized by the saturated and oxidized C ring. They are found abundantly in citrus fruits with the ability to scavenge free radicals [97].

#### 3.2.1. Hesperidin

Hesperidin is a major flavanone composed of hesperetin (aglycone) conjugated by rutinose [48]. Hesperidin is considered a potential anticancer, antioxidant, anti-depressive and immunomodulatory agent [49]. In the small intestine, hesperidin is poorly absorbed, and it is highly dependent on the conversion by the gut microbiome. Gut microbes in the large intestine cleave the attached rutinose moiety, forming hesperetin, enhancing bioavailability [50]. To investigate the impact of oral administration of hesperidin on gut microbiota composition, 100–200 mg of hesperidin was administered orally to Lewis rats for four weeks [51]. The administration of hesperidin resulted in a higher *Lactobacillus* proportion. The reported changes in the small intestine were associated with a concentration decline in monocyte chemotactic protein 1, supporting the prebiotic role of hesperidin.

#### 3.2.2. Naringenin

A natural 2,3-dihydroflavonoid, present in citrus fruits, was reported to pose multiple bioactive benefits [52]. Orally administered naringenin has a low bioavailability. Under the metabolism mediated by gut microbes, naringenin could be a precursor to several metabolites with physiological effects [53]. The effects of naringenin on commensal bacteria’s growth and genetic expressions such as *Ruminococcus gauvreauii, Bifidobacterium catenulatum* and *Enterococcus caccae* were measured using single-molecule RNA sequencing [54]. Tested bacteria responded differently to treatments of naringenin. While the upregulated genes of *Ruminococcus gauvreauii* are critical in iron uptake, the Bifidobacterium catenulatum genes are important in cellular metabolism and DNA repair. *Enterococcus caccae* downregulated genes responsible for sugar transport and upregulated transcription and protein transport pathways.

#### 3.2.3. Eriodictyol

Eriodictyol is a flavonoid present in many medicinal plants and has significant health properties [55]. Hydrolysis of eriocitrin; an antioxidant present in lemon fruits, results in the formation of eriodictyol (aglycone). Microbes involved in the bioconversion reaction in the gut include *Bacteroides distasonis* and *Bacteroides uniformis* through a O-Deglycosylation reaction [56]. Additionally, *Clostridium butyricum* further metabolize eriodictyol to 3,4-dihydroxyhydrocinnamic acid and Phloroglucinol, which poses antioxidant activities [57].

### 3.3. Isoflavones

Isoflavones are primarily found in soybeans and legumes. Isoflavones are further divided into genistein and daidzein [98]. They are known for their anticancer and DNA photoprotective effects [99].

#### 3.3.1. Genistein

Genistein is a phytosterol found abundantly in soybeans. The metabolism of genistein by gut microbiota plays a crucial role in its bioavailability and bioactivity [58]. Multiple metabolites produced by genistein bioconversion are reported, but the pathway and mechanism remain unclear. An anaerobic bacterium identified as a member of *Coriobacteriaceae* reduces the activated double bond of genistein yielding in dihydrogenistein [59]. Additionally, *E. ramulus* cleaves the C ring and degrades genistein, producing 6′-OH-O-desmethylangolensin [60,61]. More efforts are required to identify the metabolic pathways and gut enzymes involved in its bioconversion.

#### 3.3.2. Daidzein

Daidzein, is a dietary phytoestrogen abundantly found in soybeans. Structurally, it is similar to genistein, but it lacks a hydroxyl group on the fifth position. [62]. To permit the absorption of daidzein through the gut epithelium, β-glucosidase cleaves and releases sugar moiety producing the aglycone [63]. The metabolic pathway of daidzein begins with a reduction reaction yielding dihydrodaidzein through the hydrogenation of the activated double bonds, followed by O-desmethylangolensin or S- equol production depending on gut microbes. Gut bacteria involved include the *Clostridium-*like strain and *E. ramulus* [64].

### 3.4. Anthocyanins

Anthocyanins are characterized as unoxidized, unsaturated, water-soluble flavonoids found abundantly in fruits and flowers, responsible for their coloration [100]. Anthocyanins exert several health benefits such as inhibiting mutagenesis, diminishing lipid peroxidation and reducing DNA damage [101].

#### 3.4.1. Cyanidin

This flavonoid is found abundantly in crops and fruits, where it is usually conjugated to sugar [65]. Due to its low bioavailability, a substantial proportion of cyanidin ingested enters the large intestine, where it is metabolized by gut microbes [66]. Cyanidin-3- glucoside, is a metabolite degraded by gut microbes and investigated in rats [67]. Intestinal bacterial species *E. ramulus* and *Clostridium saccharogumia* convert cyanidin-3-glucoside to 2,4,6-trihydroxybenzoic acid and 2,4-dihydroxybenzoic acid via cyanidin. Cyanidin-3-glucoside metabolism was also conducted in the absence of bacterial strains, but the products were low in concentration, suggesting that bacterial conversion is critical to support the proposed beneficial effects of cyanidin-3-glucoside.

#### 3.4.2. Delphinidin

Delphinidin is an anthocyanin found profusely in berries, red cabbage, grapes and sweet potatoes [68]. Delphinidin poses anti-inflammatory, antioxidant, anti-mutagenic and anti-turmeric properties [69]. Microbial catabolism of delphinidin to gallic acid and delphinidin-3-glucoside is required to enhance bioavailability. To produce these bioactive metabolites, gut microbes such as *Lactobacillus* breaks the glycosidic linkage using intestinal gut enzymes such as β-d-glucosidase, β-d-glucuronidase, α-galactosidase and α-rhamnosidase [70,71]. Additionally, using a cyclodextrin encapsulation to enhance the stability of delphinidin, improved bioavailability, allowing their release in the colon where they are metabolized and exert their potential health benefits [72].

#### 3.4.3. Pelargonidin

It is a flavonoid found in blueberries and other berries. It displays cytotoxic effects [73]. Similar to other anthocyanins, pelargonidin requires microbial metabolism to enhance bioavailability and bioactivity. *Lactobacillus* in the colon metabolizes pelargonidin, yielding in 4-hydroxybenzoic (the absorbable form). Enzymes involved in the metabolism include β-d-glucosidase, β-d-glucuronidase, α-galactosidase and α-rhamnosidase [70]. All anthocyanins, including pelargonidin, modulate microbiota’s composition, increasing the concentration of probiotic bacteria by producing short-chain fatty acids (SCFA) [74].

### 3.5. Flavones

Flavones are widely distributed in flowers, fruits and leaves [102]. Flavones have a ketonic group at C4, an unsaturated C ring on the C2–C3, and lack hydroxylation on C3 [103].

#### 3.5.1. Baicalein

It is an aglycone present in the roots of *S. baicalensis*, with anti-neurodegenerative, anti-inflammatory and anti-cardiovascular activities [75,76]. In the intestine, baicalin is metabolized by β-glucuronidase produced by *E. coli*, yielding baicalein, thus enhancing absorption, bioavailability and bioactivity [77]. Baicalein (aglycone) poses significant anti-proliferative effects on human colon cancer cells compared to baicalin, supporting the key role of gut microbe’s bioconversion [78].

#### 3.5.2. Diosmin

A flavonoid commonly found in *Citrus* spp. It exerts a wide range of biological activities [79]. It was reported that the administration of diosmin in rats with gastric cancer resulted in the reduction of tumor rate, the suppression of inflammatory cytokines (IL-6, TNF-a and NF-KB), and the improvement of body weight [80]. Similar to other flavonoids, diosmin is poorly soluble, affecting its bioavailability. Following oral consumption, diosmin is hydrolyzed in the intestine by microbial enzymes such as α-glucosidase and β-glucosidase into its aglycone, diosmetin [81]. Bioconversion of diosmin enhances bioavailability when measured in plasma samples from healthy participants [82]. More studies are required to identify the species involved in their bioconversion.

#### 3.5.3. Apigenin

Apigenin is a phytoestrogen aglycone found abundantly in oranges, garlic, spinach, parsley and carrots [83]. The gastrointestinal tract plays a key role in the conjugation and metabolism of apigenin before entering the bloodstream [84]. Once in the colon, apigenin becomes the substrate for gut microbes that will aid in its degradation. Bacterial species capable of degrading apigenin include *Bacteroides distasonis, Eubacterium ramulus* and *Clostridium orbiscindens* yielding critical metabolites such as 3-(4-hydroxyphenyl) propionic acid [85]. 3-(4-hydroxyphenyl) propionic acid is beneficial during infection with influenzas triggering interferon type 1 pathway and preventing inflammation [86].

#### 3.5.4. Tangeretin

A flavonoid abundantly found in the citrus peel of tangerine and in citrus fruits with a reported impact on the human gut microbiome composition [87]. The administration of polymethoxyflavones (PMFs) composed of nobiletin, tangeretin and 5-demethylnobiletin as main components, significantly increased the richness of the microbial community, but not the diversity, suggesting a possible interaction between PMFs and gut microbiota [88]. *Lactobacillus* and *Bifidobacterium*, two important probiotics, significantly increased after oral administration of PMFs, indicating their beneficial health effects. Metabolism of PMFs metabolites was carried out by demethylation, hydroxylation, demethoxylation and glucuronidation in the GI tract of the mice.

#### 3.5.5. Wogonin

Wogonin is extracted from *Scutellaria baicalensis* roots and has been used as a traditional medicine as it poses both anti-bacterial and anti-viral activities [89,90]. Intestinal bacteria enzymes such as β-glucuronidase play a key role in the hydrolysis of wogonoside to its aglycone form, wogonin, facilitating its absorption and enhancing bioavailability [91]. Antibiotics administration significantly affects the plasma concentration of wogonin as observed in pseudogerm free and normal rats, suggesting that pharmacokinetics and pharmacological effects of wogonin depend heavily on the status of intestinal microbiota [92].

#### 3.5.6. Chrysin

Chrysin is an apigenin analog and is found abundantly in honey and Thai propolis [93]. After ingestion, rapid metabolism of chrysin and excretion occurs, affecting its bioavailability and rendering its beneficial effects. In the intestine, enzymes such as flavone reductase reduce chrysin to its glucuronidase and improving absorption and bioavailability [94]. Metagenomic analysis of gut microbes confirmed that *flr*-like genes are broadly distributed in the human gut microbiome [95]. More evidence is required to exclude the possibility of horizontal gene transfer in the observed gene prevalence.

## 4. Effects of Flavonoids on Gastrointestinal Cancer

The positive impact of flavonoids consumption on breast and colorectal cancer was described in our previous publications, supporting the potential role of flavonoids on GI cancer [2,104,105,106]. To evaluate this further, we looked up the effects of flavonoids on general pathways impaired by GI cancers, such as apoptosis, cellular proliferation, inflammation and invasion (Section 4.1). Additionally, we addressed the potential impact on impaired specific pathways such as P53 translocation, extracellular Signal-Regulated Kinase and integrin (Section 4.2). Table 2 summarizes the main findings reported in the literature.

### 4.1. Flavonoids Effects on Impaired General Pathways

#### 4.1.1. Apoptosis

A process described as programmed cell death is characterized by the changes in the biochemical mechanisms and the morphological characteristics of the cell [142]. It is considered a critical component of various biological processes such as embryonic development, chemical-induced cell death and normal cell turnover [143]. A wide variety of pathological and physiological stimuli and conditions could trigger apoptosis [144]. Unregulated apoptosis can result in neurodegenerative, autoimmune diseases and cancer [145]. In cancer, apoptosis is considered the hallmark for cell survival, invasiveness and cellular proliferation. There are multiple ways in which cancer cells can evade intrinsic and extrinsic apoptotic pathways, such as inhibition of caspase function and upregulation of anti-apoptotic BCL-2 proteins [146].

**Figure 3:**
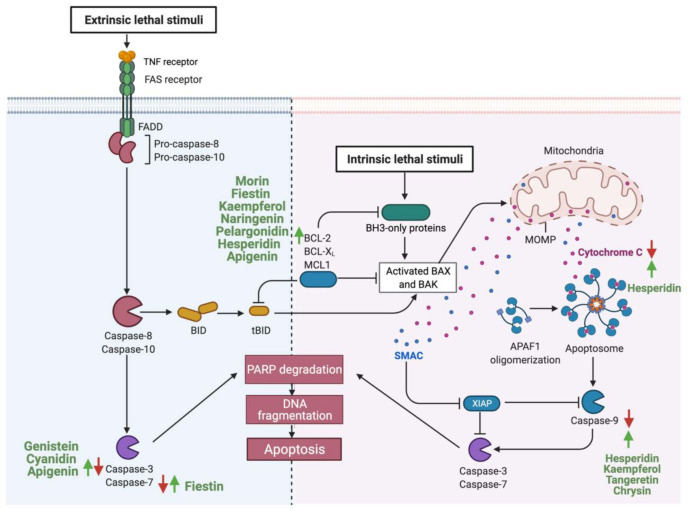
Schematic illustration of the impact of flavonoids on the apoptotic pathway. The figure is divided into two sections. In the right section, flavonoids targeting the intrinsic pathways are high-lighted, and in the left sections, the extrinsic pathways. Red arrows represent the effects of GI cancer on pathways, while the green arrows for flavonoids effect. Adapted from “Apoptosis”, by BioRender.com (accessed on 3 August 2021).

Consumption of flavonoids is reported to induce apoptosis in GI cancer, acting as a potential therapeutic agent [147]. Flavonoids could induce apoptosis by acting on the intrinsic apoptotic pathway, as in the case of fisetin. Gastric cancer cells treated with (25–100 μM) of fisetin induced apoptosis by dissipating mitochondrial potential and upregulating pro-apoptotic molecules such as Bcl-2 and tumor suppressors such as P53 [148]. Additionally, other flavonoids such as cyanidin upregulate the expression of caspase 3, therefore activating the extrinsic apoptotic pathway in human gastric adenocarcinoma cells [127]. While some flavonoids target one specific pathway, other targets both intrinsic and extrinsic pathways such as apigenin in colorectal cancer cells or, as in the case of hesperidin, flavonoids can target multiple components of the same pathway [119,135]. Figure 3 summarizes reported flavonoids that induce apoptosis of GI cancer cells and their target’s key components.

#### 4.1.2. Cellular Proliferation

Cellular proliferation is a fundamental process for homeostasis and cellular development that is tightly regulated to ensure accurate genome duplication [149,150]. Phosphatidylinositol-3-kinase (PI3K)/AKT/mammalian target of rapamycin (mTOR) pathway is one of the most critical intracellular pathways to regulate cellular survival, growth, motility and metabolism [151]. Under baseline conditions, PI3K is activated by an external stimulus such as cytokines, hormones and growth factors. Upon activation, phosphorylation yields a second messenger (PIP3) that binds and recruits lipid-binding domains that target cell membrane. Signaling proteins such as AKT kinase binds to PI3K to activate cellular growth [152]. Phosphatase and tensin homolog (PTEN) regulate the pathway by the dephosphorylation of PI3K, thus preventing downstream activation [153]. In cancer, PI3K pathway can be downregulated by the inactivation of PTEN (tumor suppressor), mutation of PI3K and activation of tyrosine kinase [154].

**Figure 4:**
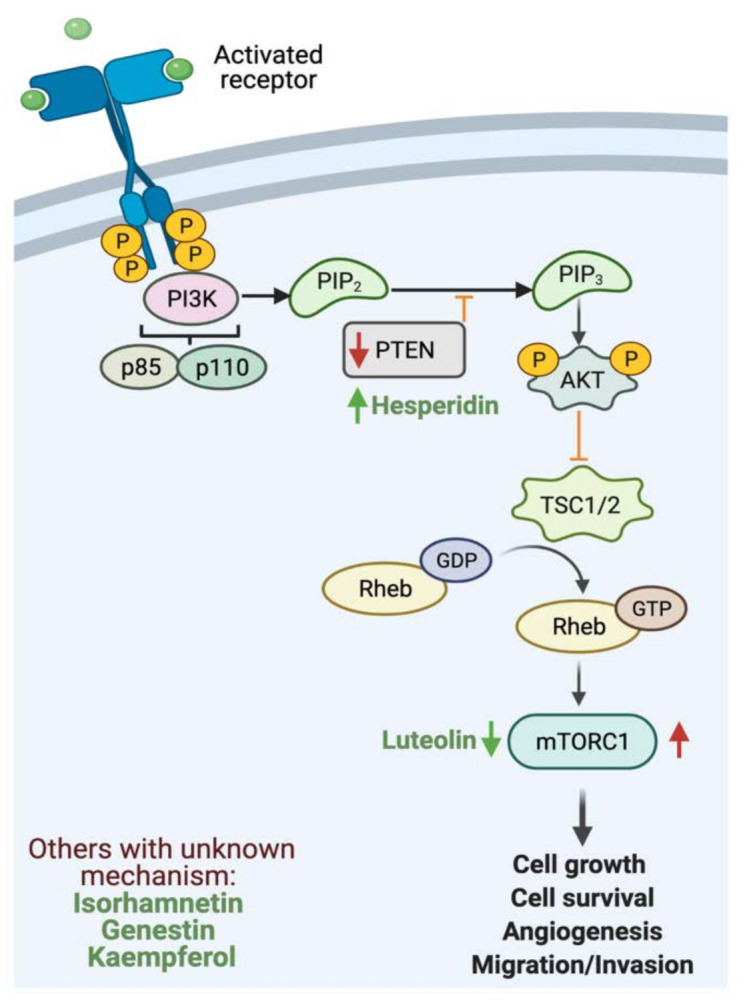
Schematic illustration of the impact of flavonoids on cellular proliferation. The figure illustrates PI3K pathway. Red arrows represent the effects of GI cancer on the pathway, while the green arrows for flavonoids effect. Adapted from “PI3K/Akt, RAS/MAPK, JAK/STAT signaling”, by BioRender.com (accessed on 3 August 2021).

Flavonoids such as hesperidin can inhibit the proliferation of cancer cells [155]. In an in vivo and in vitro study, the results provided strong evidence that hesperidin enhances antitumor effects on gastric cancer by regulating PI3K/AKT signaling pathway through the upregulation of PTEN expression. In addition, a combinatory therapy of cisplatin with hesperidin could enhance the clinical outcome [120]. Additionally, luteolin can inhibit cellular proliferation by regulating PI3K, AKT and mTOR signaling pathways, which play a key role in the progression and development of gastric cancer [132]. Figure 4 highlights the reported activities of flavonoids affecting GI cancer cells proliferation.

#### 4.1.3. Inflammation

Inflammation is a biological response of the immune system to harmful stimuli such as damaged cells, toxic compounds and pathogens [156]. Nuclear factor κB (NF-κB) is a transcription factor that regulates the immune and inflammatory responses and is considered a major proinflammatory gene function mediator [157]. Two major pathways are involved in the activation of NF-κB, the canonical and noncanonical [158]. Upon activation by stimuli, phosphorylation by multi-subunit kinase occurs to induce degradation of IκBα. Proteasomal degradation of IKB results in the rapid and transient tumor translocation of P50/RelA and P50/c Rel dimers [159]. NF-κB pathway in tumor cells is constitutively active, and its suppression inhibits the growth of tumor cells [160]. Alternative therapeutic approaches to deactivate NF-κB are required.

**Figure 5:**
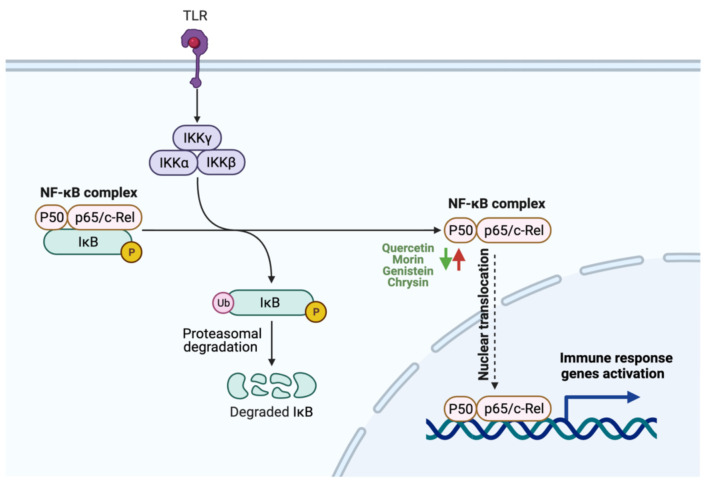
Schematic illustration of the impact of flavonoids on inflammation. The figure illustrates NF-*κ*B complex activation. Red arrows represent the effects of GI cancer on the pathway, while the green arrows for flavonoids effect. Adapted from “NF-*κ*B signaling pathway”, by BioRender.com (accessed on 3 August 2021).

Natural compounds, including flavonoids, were reported to potentially reduce inflammation in GI cancer [161,162]. The treatment of human gastric adenocarcinoma cell-line (AGS) with quercetin (10 μM) for 72 h inhibited the activation of NF-κB through the reduction of p65 phosphorylation, suggesting that quercetin can hamper NF-κB pathway activation and act as a potential anti-inflammatory agent [163]. Additionally, the administration of genistein inhibits NF-κB expression in colon cancer cells, thus reducing inflammation and suppressing tumor cell migration [164]. Moreover, chrysin administration can indirectly block NF-κB activation by suppressing Recepteur d’origine Nantais (RON), a member of c-Met family, critical for invasion in gastric adenocarcinoma [165]. Figure 5 illustrates the reported anti-inflammatory activities of selected flavonoids utilizable in GI cancer management.

#### 4.1.4. Metastasis

Cancer metastasis is the spread of cancerous cells to organs and tissues beyond the original tumor site. It is considered the leading cause of mortality in patients with cancer [166]. Five key steps in metastasis are reported: intravasation, invasion, circulation, extravasation and colonization [167]. Metastasis cascade is dependent on the loss of adhesion between cells which results in the separation from the primary tumor [168]. E- cadherin, the most abundant integral protein in epithelia, is reported to play a pivotal role in controlling cancer spread and dissociation, as tumor invasion and metastasis often coincide with the loss of E-cadherin function [169]. Additionally, E-cadherin is considered a tumor suppressor, giving its critical role in the downregulation of the epithelial-mesenchymal transition (EMT) process and the formation of proper intracellular junction [170].

Flavonoids show potent anti-cancer effects by regulating critical signaling pathways involved in cancer cells’ metastatic progression, migration, and invasion [105]. It was reported recently that chrysin and apigenin showed the synergistic capacity to inhibit the development and metastasis of colorectal cancer [171]. Indeed, flavonoids such as genistein, baicalein, luteolin and tangeretin can enhance the expression of E-cadherin in GI cancer. The effect of genistein on EMT was investigated in colon cancer using HT-29 cells [164]. At 200 μmol/L genistein could inhibit tumor migration through the upregulation of E-cadherin expression, suggesting a potential anti-metastatic agent for colon cancer.

**Figure 6:**
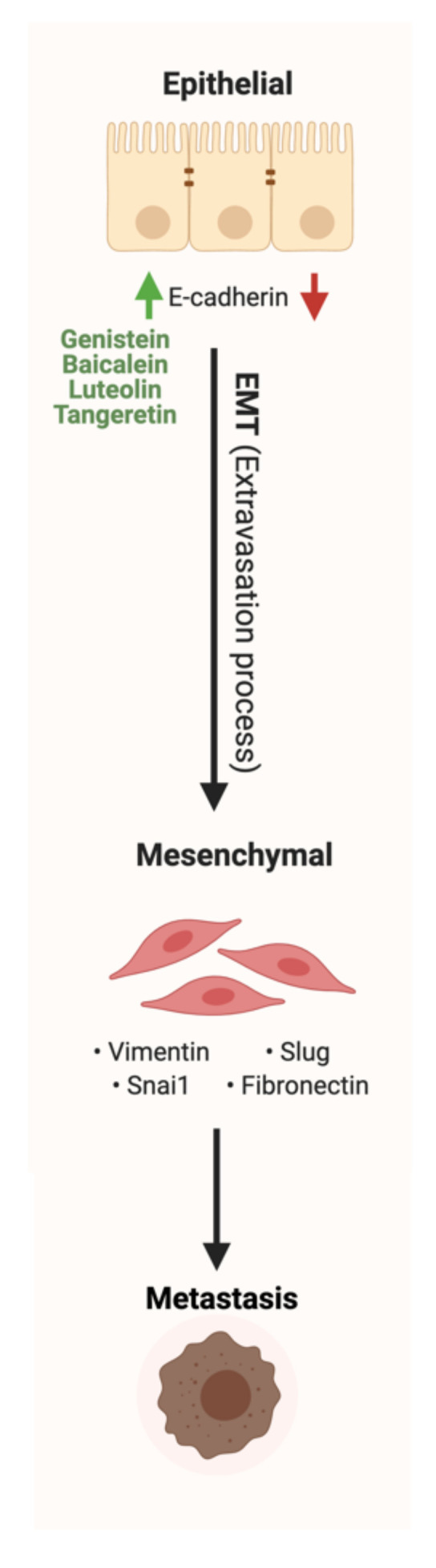
Schematic illustration of the impact of flavonoids on metastasis and EMT pathway. Red arrows represent the effects of GI cancer on the pathway, while the green arrows illustrate the impact of flavonoids on the pathway. Created with BioRender.com (accessed on 3 August 2021).

Additionally, using ultra-high-performance liquid chromatography, baicalein reduced the expression of EMT and enhanced E-cadherin expression in colon cancer cells [130]. In addition, gastric cancer metastasis and progression were inhibited significantly after the administration of luteolin and tangeretin [137,172]. Luteolin could reverse EMT expression through the induction of E-cadherin expression, and the downregulation of Snail and vimentin, and tangeretin reduced the expression of EMT. Figure 6 represents a schematic illustration of the reported anti-metastatic effects of flavonoids in GI cancers.

### 4.2. Flavonoids Effects on Impaired Specific Pathways

**Figure 7:**
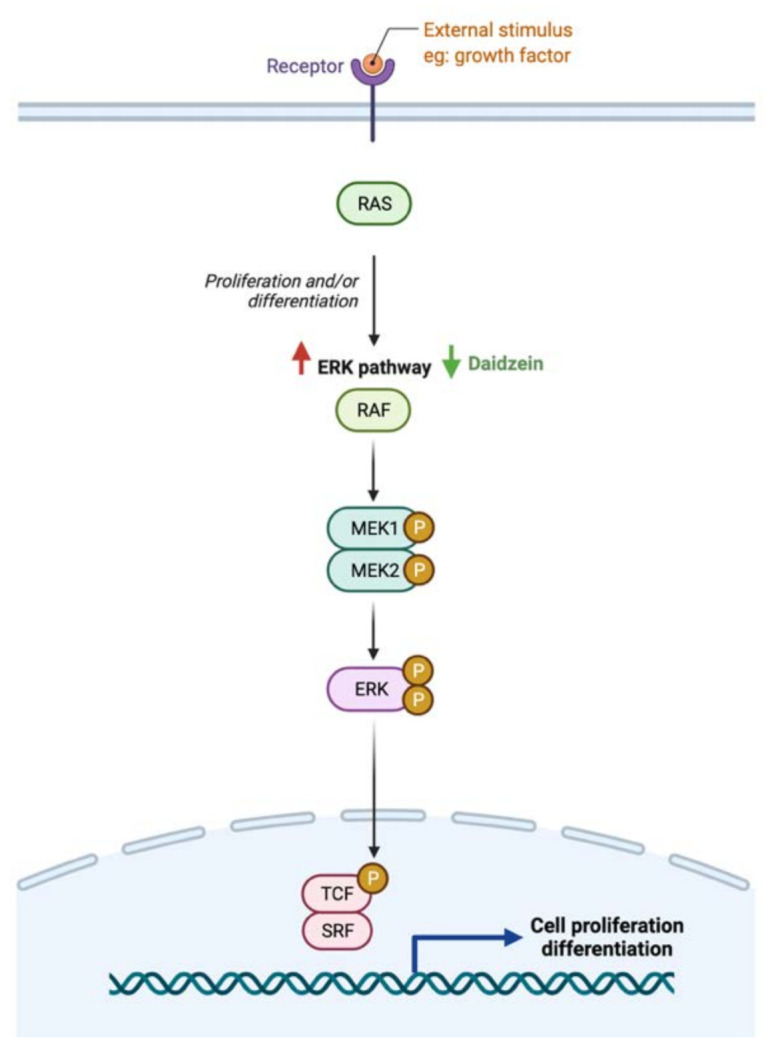
Schematic illustration of the impact of flavonoids on ERK pathway. Red arrows represent the effects of GI cancer on the pathway, while the green arrows illustrate the impact of flavonoids on the pathway. Created with BioRender.com (accessed on 3 August 2021).

**Figure 8:**
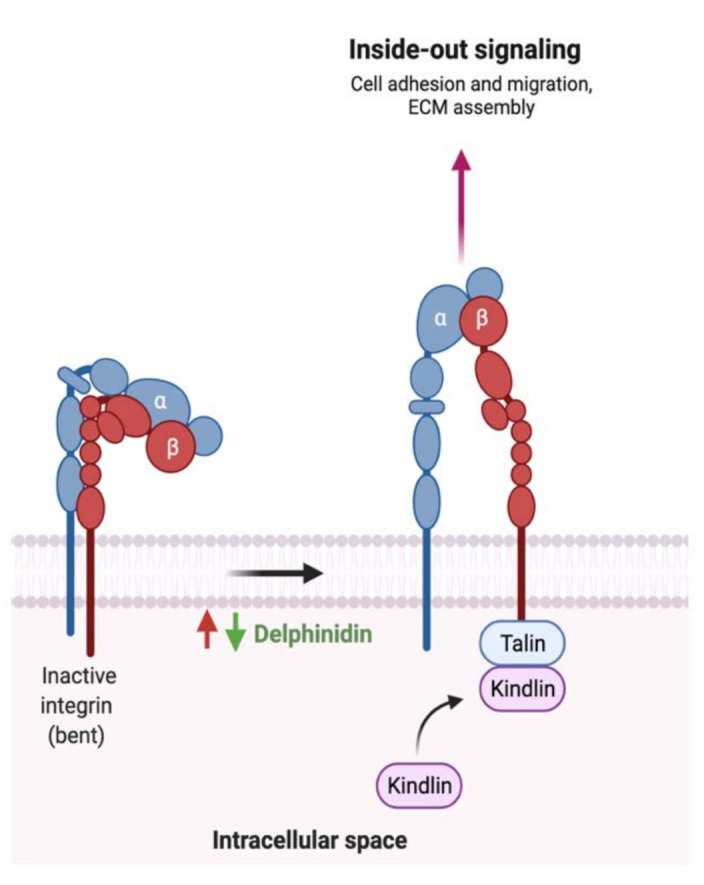
Schematic illustration of the impact of flavonoids on integrin activation. Red arrows represent the effects of GI cancer on the pathway, while the green arrows illustrate the impact of flavonoids on the pathway. Adapted from “Outside-in and Inside-out Integrin Signaling Pathways”, by BioRender.com (accessed on 3 August 2021).

**Figure 9:**
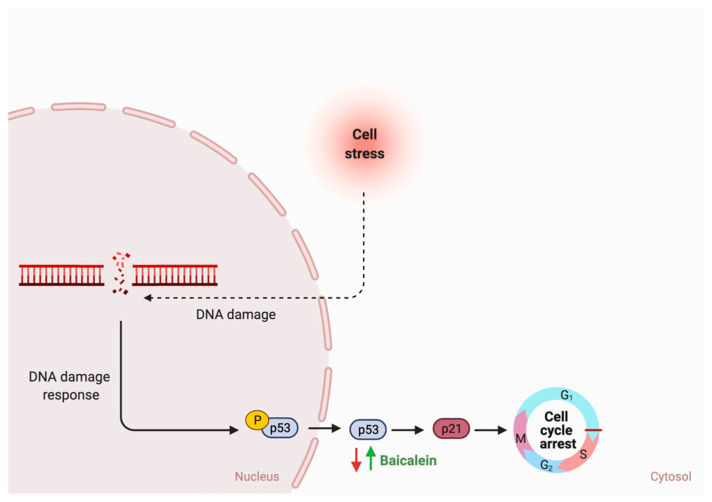
Schematic illustration of the impact of flavonoids on cell cycle arrest. Red arrows represent the effects of GI cancer on the pathway, while the green arrows illustrate the impact of flavonoids on the pathway. Created with BioRender.com (accessed on 3 August 2021).

The literature review revealed that flavonoids target and positively affect three pathways in GI cancer. Diazedine could inhibit choriocarcinoma cellular proliferation through the suppression of the extracellular-signal-regulated kinase (ERK) pathway by inhibiting p-ERK expression and translocation, as illustrated in Figure 7 [126]. Additionally, Figure 8 illustrates the mechanisms in which the metastasis of colon cancer was inhibited after the administration of dietary delphinidin. Treatment of colon cancer cells lines such as sw480 with delphinidin (<100 μM) suppressed the invasiveness of cell lines and downregulated the integrin signaling pathway, critical for cell adhesion and migration [128]. Moreover, baicalein administration on cells undergoing stress could result in cellular cycle arrest through the activation of p53, as shown in Figure 9 [173]. More efforts are required to understand the underlying mechanism and the potential of using them in cancer therapy.

## 5. Discussion

### 5.1. Clinical Implementation of Flavonoids

As illustrated in Section 4, flavonoids implementation in GI cancer treatments has tumor-suppressive activities in theory. To effectively use flavonoids in cancer therapy, clinical trials are required to assess the impact of flavonoids subclass on GI cancer and gut enzymes. In 2003, flavopiridol, a synthetic flavone reported to inhibit cell cycle progression, was evaluated in 20 patients with advanced colorectal cancer. The patients received flavopiridol at a dose of 50 mg/m^2^/day every 14 days via continuous infusion for eight weeks. The phase II clinical trial results reported minimal hematological activities and moderate diarrhea and fatigue in the 20 patients. Even though the pre-clinical data showed promising antitumor activities, no impartial response was observed as only 28% of the patients experienced stabilization of the disease [174]. Limited studies are reported in the literature, and more are required to evaluate the appropriate dose-administered, the appropriate stage of the disease that tolerates flavonoids administration, and the impact of combination therapy (flavonoids and chemotherapy or a mixture of flavonoids) have synergistic effects on gut enzymes and GI cancer.

### 5.2. Impact of Current Cancer Treatment on Gut Enzymes

The field of modern oncology has produced significant advances in cancer treatment, improving and prolonging patient’s lives [175]. Due to observed long-term side effects on cancer survivors, cancer microbiome research that addresses the crucial role of the gut microbiome in improving the efficacy of cancer therapy is rapidly emerging [176]. Chemotherapy can have devastating effects on microbial diversity leading to gastrointestinal toxicities, acute dysbiosis and delaying the response to treatment. Modulation of the gut microbiome was suggested as a practical therapeutic approach to improving cancer treatment’s toxic side effects [177]. *Lactobacillus* and *Bifidobacterium* along with one digestive enzyme were evaluated for their efficacy in protecting the GI tract after chemotherapy treatment. The results showed an improvement in the colon’s fermentation process and the recovery of microbial population in which the ratio of *Bacteroidetes* to *Firmicutes* was restored, inducing microbial metabolites production and enhancing anti-inflammatory response [178].

Moreover, a combination of rutin and chemotherapeutic agent Oxaliplatin promoted apoptosis of gastric cancer cells SGC-7901 demonstrated through decreased BCL-2/Bax ratio while the apoptotic mechanisms of rutin were related to caspase-mediated signaling [179]. In addition, luteolin combined with Oxaliplatin, suppressed proliferation and induced apoptosis in gastric cancer SGC-7901 cells through cleaved caspase-3, upregulated Bax and downregulated Bcl-2 [133]. More efforts are required to address the impact of cancer treatment on gut enzymatic activities (enhancement/depletion) and natural product metabolism.

### 5.3. Fecal Transplantation: Could It Be the Solution?

Fecal microbiota transplant (FMT) is defined as the transplantation of a fecal solution from a selected donor into the GI tract of a patient to change and restore microbial composition posing as a powerful and effective therapy method [180]. In cancer therapy, laboratory mice transplanted with intestinal microbes from wild mice reported better colorectal cancer resistance than the control [181]. Even though FMT presents a promising therapeutic strategy, efforts are required to identify its safety and efficacy, possible side effects and the possibility of restoring gut enzyme activities required for flavonoids metabolism. Furthermore, additional efforts are needed to propose a strategy where current cancer treatments and FMT could be used, resulting in a synergistic and compelling effect.

### 5.4. Challenges with Studying the Field

The microbiome field has received attention over the last 15 years; however, putative key issues and misinterpretation still pose a problem. Efforts are required to identify the appropriate level to investigate microbial composition (phylum, genus, species). Additionally, microbes in the gut are in a complex community. Therefore, identifying a single microorganism and correlating it to a particular disease without supporting evidence may negatively impact the field. The gut microbiome composition is influenced by multiple factors such as genetic, environment, lifestyle, diet and geographical distribution [182]. Developing a tool or a strategy to standardized experimental activities to allow generalized results interpretation is a necessity.

### 5.5. Estimated Flavonoid Concentration and Anti-Cancer Effects

The total consumption level of flavonoids in a day is reported to range from 11 to 600 mg depending on geographical location, food processing methods, flavonoids solubility and dietary habits [12]. In most of the reviewed studies and based on the flavonoids tested, method of purification, model and cell lines used and the method of inoculation, variations in the concentration were observed. For example, in a study evaluating the impact of rutin on cellular proliferation in colorectal cancer, the concentration used to achieve the significant proliferation reduction ranged from 31.25 to 1000 μM (72 h study period) [107]. On the other hand, quercetin induced apoptosis in gastric cancer cells with a concentration ranging from 3.125 to 400 μM (48 h study period) [113]. Even though both studies purchased their tested flavonoids (purity not mentioned), and both flavonoids belong to the same flavonoid classification (flavonol), the concentration required to achieve the anti-cancer effects varied. More efforts are required to standardize the isolation and purification method, recommend dosage required for each cancer, and understand the effects of flavonoids bioavailability, solubility and metabolic alteration on the observed anti-cancer effects.

### 5.6. Possible Synergistic Effects of Flavonoids?

While the type and concentration of flavonoids consumed are critical to observe the possible biological activities, selecting the ones that trigger multiple metabolic pathways may be improve the pathogenesis of GI cancer. For instance, genistein, an isoflavone, triggers four different pathways in cancer such as the extrinsic apoptotic pathway by activating caspase 3 activates, cellular invasion by enhancing the expression of E- cadherin, cellular proliferation by an unknown mechanism, and cellular inflammation by reducing nuclear translocation of NF-κB. On the other hand, baicalein, delphinidin and daidzein act on more specific pathways (Figure 7, Figure 8 and Figure 9). By combining these four flavonoids together, could their anti-cancer effects improve as they targeted multiple pathways? Moreover, could they complement each other pathway-wise? Currently, these are only suggestions that need further research to support and avoid possible side effects. Also, more research is needed to investigate the mechanism of hesperidin, a flavanone, which triggers both intrinsic and extrinsic apoptotic pathways.

## 6. Conclusions

Flavonoids ubiquitously found in fruits and vegetables are metabolized in the body by gut enzymes and pose a potential therapeutic and preventive tool to reduce the risk of GI cancer. Generally, signature bacteria in the gut with specific enzymatic activities that produce flavonoid metabolites could enhance the anticancer effects by triggering multiple pathways. Despite that, more research is required to identify the major enzymes, the impact of current treatments on enzymatic activities and the appropriate flavonoids dose required to achieve the observed anticancer effects. Additionally, clinical trials assessing those benefits are needed to estimate the potential of using flavonoids in a clinical setting against cancer.

## Figures and Tables

**Table 1:** Representative Flavonoids and their Underlying Metabolism by Gut microbiota.

Flavonoid Subclass	Name of Flavonoid	Structure of Flavonoid	Dietary Source	Metabolites Produced by Gut Microbiota	Bacteria Involved in Metabolism	Enzymes Involved in Metabolism	Site of Metabolism	Conversion Mechanism		Effect of Microbiota on Flavonoids	Model Used		References
											In Vivo	In Vitro	
Flavonol	1. Rutin	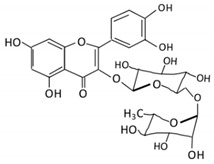	Lemons, berried, limes and oranges	Quercetin -3- O- glucoside Quercetin	*Lachnoclostridium Eisenbergiella* *Escherichia* *Parabacteroides* *Erysipelatoclostridium*	α-rhamnosidases β-glucosidases	Colon	Hydrolysis of rutin to remove sugar moiety		Permit the absorption of the aglycone Enhance bioavailability		10 fecal samples from healthy individual following omnivore diet.	[29,30,31,32,33]
	2. Fisetin	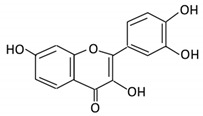	Persimmon and onions	No available data									[34,35]
	3.Kaempferol	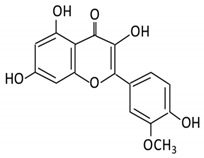	Tea, berries and cruciferous vegetables	Kaempferol -3-O- glucoside p-coumaric acid kaempferol 3-(4 hydroxyphenyl) propionic acid 3-phenylpropionic acid	*Lactobacillus* *paracasei* A221	β-glucosidases	Intestinal tract	(A)Degradation through multiple chemical reactions: - Deglycosylation - Reduction - Dehydroxylation (B) Hydrolysis		Permit the absorption of the aglycone Enhance bioavailability	Mice	3 fecal samples from healthy individual Caco-2 intestinal barrier model	[36,37,38,39]
	4. Quercetin	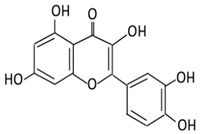	Black currants, cherries, apples and chokeberries	4-hydroxybenzoic acid 3,4-dihydroxyphenylacetic acid 3,4-dihydroxybenzoic 3-(3-hydroxyphenyl) propionic acid	*Bacteroides fragilis* *Clostridium perfringens* *Eubacterium ramulus* *Streptococcus S-2 Lactobacillus L-2* *Bifidobacterium B-9 Bacteroides JY-6*	Lactate phlorizin hydrolase	Small intestine	Deglycosylation		Enhance bioavailability	Mice	87 fecal samples from healthy elderly individual	[40,41,42,43,44,45]
	5. Isorhamnetin	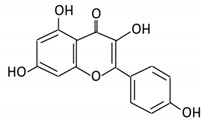	Ginkgo biloba and Hippophae rhamnoides	3-O-neohesperidoside Isorhamnetin -3- glucoside Aglycone isorhamnetin	*Escherichia* *Enterococcus* *Bacillus.*	Not identified	Small intestine	Deglycosylation		Permit the absorption of the aglycone	Rats	1 fecal sample from healthy female	[46,47]
	6. Morin	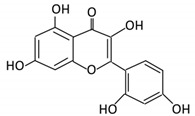	Psidium guajava, Prunus dulcis (Almond), chlorophora tinctoria and fruits	Morin glucuronides Morin sulfates	No available data								[12]
Flavanones	7. Hesperidin	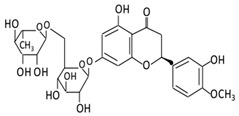	Orange citrus aurantium	Hesperetin	Not identified	Rutinose	Large intestine	Cleaves the attached rutinose moiety	Permit the absorption of the aglycone Enhance bioavailability			fecal/ blood samples from 18 Lewis male rats	[48,49,50,51]
	8. Naringenin	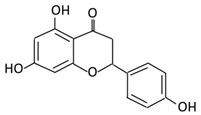	*C.aurantium* (chinese herbs) and grapefruit	Aglycone naringenin	*Ruminococcus gauvreauii* *Bifidobacterium* *catenulatum* *Enterococcus caccae* *Eubacterium ramulus*	Chalcone isomerase	Large intestine	Remove sugar group	Permit the absorption of the aglycone Enhance bioavailability			fecal samples from healthy individuals	[52,53,54]
	9. Eriodictyol	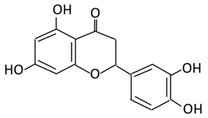	Lemon (Eriocitrin), Torr, Eridictyon californicum,	Eriodictyol 3,4-dihydroxyhydrocinnamic acid Phloroglucinol	*Parabacteroides* *distasonis* *Bacteroides uniformis JCM 5828*	Chalcone isomerase	Colon	O-Deglycosylation	Permit the absorption of the aglycone Enhance bioavailability			fecal samples from healthy individuals	[55,56,57]
Isoflavones	10. Genistein	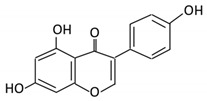	Fava and soy beans	Dihydrogenistein 6-hydroxy-O-desmethylangolensin 2-(4-hydroxyphenyl) propionic acid	*Lactobacillus* *Eubacterium ramulus*	Lactate phlorizin hydrolase	Small intestine	Fermentation by anerobic bacteria	Permit the absorption of the aglycone Enhance bioavailability			Samples from C57BL/6 female mouse	[58,59,60,61]
	11. Daidzein	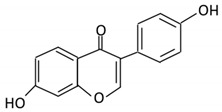	Soybeans, nuts and soymilk	Dihydrodaidzein O-desmethylangolensin S- equol	*Clostridium-*like strain	β- glucosidase Lactate phlorizin hydrolase	Colon	Reduction	Permit the absorption of the aglycone Enhance bioavailability		Mice		[62,63,64]
Anthocyanins	12. Cyanidin	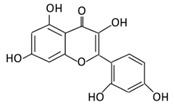	Bilberry, blueberry, grapes, blackberries, hawthorn	Cyanidin-3- glucoside	*Clostridium saccharogumia* *Eubacterium ramulus*	Combined activity of bacteria and host enzymes	Small intestine	Degrade polyphenolic glycosides	Permit the absorption of the aglycone Enhance bioavailability		Dawley rats	Fecal samples from healthy individuals	[65,66,67]
	13. Delphinidin	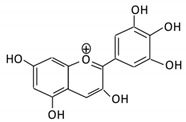	Dark grapes, eggplant, berries, red cabbage,	Gallic acids	*Lactobacillus*	β-d-glucuronidase β-d-glucosidase α-rhamnosidase α-galactosidase	Colon	Cleavage of glycosidic bonds	Permit the absorption of the aglycone Enhance bioavailability		Mice		[68,69,70,71,72]
	14. Pelargonidin	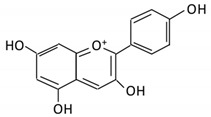	Bilberry and *ficus bengalensis* Linn	4-hydroxybenzoic	*Lactobacillus*	β-d-glucosidase β-d-glucuronidase α-galactosidase α-rhamnosidase	Colon	Cleavage of glycosidic bonds	Permit the absorption of the aglycone Enhance bioavailability		Mice		[73,74]
Flavones	15. Baicalein	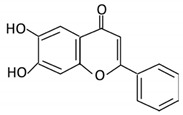	*Scutellaria lateriflora L*	Baicalein	*E. coli*	β-glucuronidase	Intestine	Hydrolysis to remove moiety	Permit the absorption of the aglycone Enhance bioavailability		Mice	HCT-166 cells SW-480 cells	[75,76,77,78]
	16. Luteolin	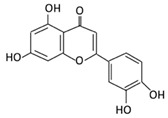	broccoli, celery and parsley,	No available data									[12]
	17. Diosmin	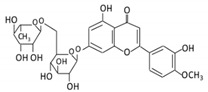	Citrus fruits	Diosmetin	Not identified	α- glucosidase β- glucosidase	Small intestine	Hydrolysis	Enhance bioavailability			Blood samples from healthy participants	[79,80,81,82]
	18. Apigenin	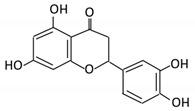	Tea, chamomile, parsley and oranges	3-(4-hydroxyphenyl) propionic acid Apigenin	*Bacteroides distasonis* *Eubacterium ramulus* *Clostridium* *orbiscindens*	β- glucosidase lactase-phlorizin hydrolase	Small intestine	Glucuronidation Hydrolysis	Enhance bioavailability		Rats	Fecal and urine samples	[83,84,85,86]
	19. Tangeretin	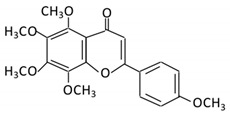	*Poncirus trifoliate L,* citrus fruit	Tangeretin-O-glucuronides	*Lactobacillus* *Bifidobacterium*	Possibly by: A) rhamno glucosides B) C- glycosyl	Small intestine	Demethylation	Permit the absorption of the aglycone		Rats		[87,88]
	20. Wogonin	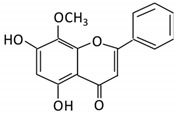	*Scutellaria baicalensis* Georgi	Wogonin	Not identified	β-glucuronidase	Intestine	Hydrolysis	Enhance absorption and bioavailability		Sprague-Dawley rats		[89,90,91,92]
	21. Chrysin	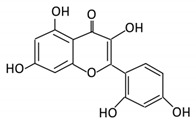	Honey	Chrysin glucuronides	*Flavonifractor plautii ATCC 49531*	Flavone reductase	Intestine	Reduction	catalyzes the hydrogenation of the C2–C3 double bond			ATCC 49,531 strain	[93,94,95]

**Table 2:** Representative Flavonoids and their Underlying Anticancer Effects.

Flavonoid Subclass	Name of Flavonoid	Metabolites Produced by Gut Microbiota	Type of Cancer (s)	Targeted Pathways	Mechanism of Action	Methods of Testing	Model Used		References
							In Vivo	In Vitro	
Flavonol	1. Rutin	Quercetin -3- O- glucoside Quercetin	Colorectal cancer Colonic adenocarcinoma	Reactive oxygen species (ROS) Apoptosis Angiogenesis	Inhibit tumor cell proliferation Impaired attachment of cells in dose-dependent matter (38 ± 1.9%) Inhibit cell migration Protect cells from inflammation, oxidative stress, and DNA damage	Cell viability assay Cell migration assay Adhesion assay Haptotaxis assay	Azoxymethane (AOM) treated mice	Caco-2 cells HT29 cells	[107,108,109]
	2. Fisetin	No available data	Gastric cancer Colorectal cancer	Apoptosis Caspase -7 B-cell lymphoma 2 (Bcl-2)	Reduce cellular proliferation significantly Reduce anti-apoptotic proteins Reduce the activation of extracellular- signal-regulated kinase ½ in dose- dependent matter Increase the proportion of cells at G2/M phase Increase the level of caspase 7 Induce cellular apoptosis (87% after treatment)	Flow cytometry Western blot Cell counting kit-8 assay Immunoblotting	Apc ^Min/+^ males Wistar rats	SGC7901 cells PIK3CA cells	[110]
	3. Kaempferol	Kaempferol -3-O- glucoside p-coumaric acid kaempferol 3-(4 hydroxyphenyl) propionic acid 3-phenylpropionic acid	Gastric cancer Colorectal cancer	B-cell lymphoma 2 (Bcl-2) Protein kinase B (Akt) Cyclooxygenase-2	Inhibit cellular proliferation significantly Inhibit the growth of tumor xenograft Reduce the expression level of cyclin B, Cyclin-dependent kinase 1 and cdc25c phosphatase Reduce the expression of Bcl-2 Increase the proportion of cells at G2/M phase Increase the level of caspase 3 and 9 * Induce cellular apoptosis	Cell viability assay Western blot Tumor xenograft experiment Ki-67 immunochemistry	Mice	SGC7901 cells MKN28 cells	[111,112]
	4. Quercetin	3,4-dihydroxyphenylacetic acid 3-(3-hydroxyphenyl) propionic acid 3,4-dihydroxybenzoic 4-hydroxybenzoic acid	Gastric cancer	Nuclear factor kappa (NF-κB) Protein kinase B (Akt) AMP-activated protein kinase	Inhibit NF-κB, AMPKA pathways Reduce matrix metalloproteinases Reduce cellular migration and invasion Reduce tumor metastasis Induce cellular apoptosis	Cell viability assay Western blot Flow cytometry Quantitative reverse transcription PCR	Mice	AGS cells GCBGC823 cells MGC803 cells	[113,114]
	5. Isorhamnetin	3-O-neohesperidoside Isorhamnetin -3- glucoside Aglycone isorhamnetin	Gastric cancer Colorectal cancer	Protein kinase B (Akt) Phosphoinositide 3-kinase Peroxisome proliferator-activated receptor gamma (PPAR-γ)	Inhibit cellular proliferation Reduce the expression of anti-apoptotic proteins Reduce cellular invasion Induce cellular apoptosis Increase PPAR-γ expression	PPAR-γ competition assay Flow cytometry Western blot Invasion assay	Mice	AGS cells MKN45 cells	[115,116]
	6. Morin	Morin glucuronides Morin sulfates	Colorectal cancer	Peroxisome proliferator-activated receptor gamma (PPAR-γ) Nuclear factor kappa (NF-κB) B-cell lymphoma 2 (Bcl-2)	Inhibit cellular proliferation in dose and time dependent matter Inhibit NF-κB, AMPKA pathways Reduce glucose uptake Reduce antioxidant activities Reduce ATP production level Induce cellular apoptosis Increase PPAR-γ expression	Cell viability assay Flow cytometry Immunoblotting Antioxidant assay		SW480 cells	[117,118]
Flavanones	7. Hesperidin	Hesperetin	Gastric cancer	Mitochondrial pathway (increase ROS) B-cell lymphoma 2 (Bcl-2) Cytochrome c specificity protein 1 (Sp1) Phosphatase and tensin homolog	Inhibit cellular proliferation in dose and time dependent matter Inhibit xenograft tumor Reduce the expression of Bcl-2 Supress mRNA/protein level of Sp1 Reduce ATP production level Induce cellular apoptosis through PTEN expression Increase cytochrome c, caspase 3 and 9 expression	Cell viability assay Western blot Transwell invasion assay Antibody studies	Mice	SGC-7901cells MGC-803 cells HGC-27 cells	[119,120]
	8. Naringenin	Aglycone naringenin	Gastric cancer Colorectal cancer	B-cell lymphoma 2 (Bcl-2) Cyclin D1 protein Prostaglandin-endoperoxide synthase 2 (COX-2)	Inhibit cellular proliferation in dose and time dependent matter Reduce cellular migration and invasion Reduce the expression of Bcl-2 Reduce cyclin D1 protein Induce cellular apoptosis Increase the level of cleaved caspase	Quantitative reverse transcription PCR Western blot Proliferating cell nuclear antigen (PCNA)	Azoxymethane (AOM) treated rats Wistar rats	SGC-7901cells SW480 cells HCT-116 cells	[121,122]
	9. Eriodictyol	Eriocitrin	Colorectal cancer	β-glucuronidase Lipid peroxidation Superoxide dismutase (SOD) catalase (CAT)	Supress cellular proliferation Reduce lipid peroxidation levels Reduce aberrant crypt foci Reduce microbial enzymatic activities in feces Reduce Argyrophilic nucleolar organizer region (AgNOR) Induce cellular apoptosis Increase the level of cleaved caspase	AgNOR staining Bacterial enzymes assay Lipid peroxidation Histopathological assays	Albino wistar rats		[123]
Isoflavones	10. Genistein	Dihydrogenistein 6-hydroxy-O-desmethylangolensin 2-(4-hydroxyphenyl) propionic acid	Gastric cancer Colorectal cancer	Protein kinase B (Akt) Notch1 signaling E-cadherins Nuclear factor kappa (NF-κB)	Blocked high cell migration capacity of CD44 Reduce Glil and CD44 expression Supress cellular invasion and metastasis Decrease epithelial-mesenchymal transition (EMT) expression Inhibit Notch1 and NF-κB expression Induce cellular apoptosis Increase E-cadherins and caspase 3 expression	Colony formation assay Western blot Flow cytometry Real time polymerase chain reaction Immunofluorescence		MKN 45 cells HT-29 cells HCT-116 cells	[124,125]
	11. Daidzein	Dihydrodaidzein O-desmethylangolensin S- equol	Colorectal cancer Choriocarcinoma	Peroxisome proliferator-activated receptor gamma (PPAR-γ) Extracellular-signal-regulated kinase (P-ERK)	Reduce lipid droplet accumulation Reduce Phosphoinositide 3-kinase (PI3K) expression Inhibit cellular proliferation Reduce the expression of P-ERK Induce cellular apoptosis Induce m-RNA expression of PPAR-γ	Colony formation assay Western blot Flow cytometry Real time polymerase chain reaction Immunofluorescence	Nude mice	JAR cells JEG-3 cells HT-29 cells	[126]
Anthocyanins	12. Cyanidin	Cyanidin-3- glucoside	Gastric adenocarcinoma	Combined activity of bacteria and host enzymes	Reduce cellular proliferation Induce cellular apoptosis through caspase-3 activation	Cell viability assay High-performance liquid chromatography (HPLC)		AGS cells	[127]
	13. Delphinidin	Gallic acids	Colorectal cancer	αV/β3-integrin miR-204-3p	Inhibit colony formation Reduce cellular viability (not significant) Inhibit migration and invasion through αV/β3-integrin reduction and miR-204-3p enhancement	Colony formation assay Western blot Flow cytometry Microarray	BALB/C nude mice	SW480 cells SW620 cells	[128]
	14. Pelargonidin	4-hydroxybenzoic	Colorectal cancer	Apoptotic pathway	Reduce the expression of Bcl-2 Induce cellular apoptosis through intrinsic pathway Induce the loss of mitochondrial membrane potential	Cell viability assay Western blot		HT-29 cells	[129]
Flavones	15. Baicalein	Baicalein	Gastric cancer Colorectal cancer	Inhibitory cell cycle proteins E-cadherins epithelial-mesenchymal transition (EMT)	Supress cellular proliferation, migration and invasion in dose and time dependent matter Decrease the expression of EMT Induce cellular apoptosis Increase the expression of E- cadherins Increase the level of cell cycle inhibitory proteins (P53 and P21)	Flow cytometry Western blot Cell counting kit-8 assay Transwell assay Colony formation assay Real time polymerase chain reaction	Mice	SGC-7901cells SW480 cells HCT-116 cells HT-29 cells	[130,131]
	16. Luteolin	No available data	Gastric cancer Colorectal cancer	E-cadherins epithelial-mesenchymal transition (EMT) Protein kinase B (Akt) Mechanistic target of rapamycin (mTOR) Notch1 signaling	Reduce cellular viability in time and dose dependent matter Inhibit cellular proliferation, migration and invasion Reduce aberrant crypt foci Decrease the expression of EMT by increasing E-cadherins and decreasing Notch1 signaling Induce cellular apoptosis	Flow cytometry Western blot Cell viability assay Xenograft assay	Mice	SGC-7901cells MKN 45 cells BGC-832 cells	[132,133]
	17. Diosmin	Diosmetin	Colorectal cancer	No available data	Inhibit aberrant crypt foci Reduce cellular proliferation	Histopathological assays Microscopic evaluation	Azoxymethane (AOM) treated rats		[134]
	18. Apigenin	Apigenin 3-(4-hydroxyphenyl) propionic acid	Gastric cancer Colorectal cancer	β-catenin B-cell lymphoma 2 (Bcl-2)	Reduce mitochondrial membrane potential Inhibit cellular proliferation Reduce the expression of Bcl-2 Increase the expression of caspase 3 Induce cellular apoptosis through intrinsic and extrinsic pathways	Flow cytometry Western blot Cell viability assay Real time polymerase chain reaction		SGC-7901cells HCT-116 cells HGC-27 cells	[135,136]
	19. Tangeretin	Tangeretin-O-glucuronides	Gastric cancer	Notch1 signaling	Reduce cellular viability, invasion and migration Inhibit Notch1 signaling pathway Decrease the expression of EMT by increasing E-cadherins Increase caspase 3 and 9 expression Induce cellular apoptosis through intrinsic and extrinsic pathways	Flow cytometry Western blot Cell viability assay Microarray	Nude mice	AGS cells MKN-45 cells MGC 80-3 cells	[137,138]
	20. Wogonin	Wogonin	Gastric cancer Colorectal cancer	P53 translocation	Reduce tumor multiplicity Induce cellular apoptosis through increase endoplasmic reticulum stress	Flow cytometry Western blot Cell viability assay Real time polymerase chain reaction	Azoxymethane (AOM) treated mice	HCT-116 cells	[139,140]
	21. Chrysin		Gastric cancer Colorectal cancer	Nuclear factor kappa (NF-κB) SALL-4 Early growth response 1 (Egr1)	Reduce tumor volume by downregulating SALL-4 expression Reduce cellular proliferation Supress the expression of NF-κB and Egr1 Increase caspase 3 and 9 expression Induce cellular apoptosis through intrinsic pathway (caspase 3 and 9)	Flow cytometry Western blot Cell viability assay Quantitative reverse transcription PCR Apoptotic assays	BALB/mice	HT-29 cells CT-26 cells	[141]

## Abbreviations

GI	gastrointestinal
CRC	colorectal cancer
GC	gastric cancer
EC	esophageal cancer
HCC	hepatocellular carcinoma
PC	pancreatic cancer
PI3K	phosphatidylinositol 3-kinase
AKT	protein kinase B
NF-κB	nuclear factor kappa
EMT	epithelial-mesenchymal transition
*E. ramulus*	*Eubacterium ramulus*
HLPC	High-performance liquid chromatography
FISH	Fluorescence in situ hybridization
PMFs	polymethoxyflavones
BCL-2	B-cell lymphoma 2
PTEN	Phosphatase and tensin homolog
RON	d’origine Nantais
ERK	extracellular-signal-regulated kinase
ROS	Reactive oxygen species
PPAR-γ	Peroxisome proliferator-activated receptor gamma
Sp1	specificity protein 1
COX-2	Prostaglandin-endoperoxide synthase 2
SOD	Superoxide dismutase
mTOR	Mechanistic target of rapamycin
Egr1	Early growth response 1
FMT	Fecal microbiota transplant
MEK	Mitogen-activated protein kinase kinase
TCF	T cell factor
IKK	IκB kinase
